# The Role of Oxidative Stress in Intervertebral Disc Degeneration

**DOI:** 10.1155/2022/2166817

**Published:** 2022-01-12

**Authors:** Guoshuai Cao, Sidong Yang, Jianye Cao, Zixuan Tan, Linyu Wu, Fang Dong, Wenyuan Ding, Feng Zhang

**Affiliations:** ^1^Department of Clinical Medicine, Hebei Medical University, Shijiazhuang 050051, China; ^2^Department of Spine Surgery, The Third Hospital of Hebei Medical University, Shijiazhuang 050051, China; ^3^Department of Rehabilitation Medicine, The Third Hospital of Hebei Medical University, Shijiazhuang 050051, China; ^4^Department of Clinical Laboratory Medicine, The Third Hospital of Hebei Medical University, Shijiazhuang 050051, China

## Abstract

Intervertebral disc degeneration is a very common type of degenerative disease causing severe socioeconomic impact, as well as a major cause of discogenic low back pain and herniated discs, placing a heavy burden on patients and the clinicians who treat them. IDD is known to be associating with a complex process involving in extracellular matrix and cellular damage, and in recent years, there is increasing evidence that oxidative stress is an important activation mechanism of IDD and that reactive oxygen and reactive nitrogen species regulate matrix metabolism, proinflammatory phenotype, autophagy and senescence in intervertebral disc cells, apoptosis, autophagy, and senescence. Despite the tremendous efforts of researchers within the field of IDD pathogenesis, the proven strategies to prevent and treat this disease are still very limited. Up to now, several antioxidants have been proved to be effective for alleviating IDD. In this article, we discussed that oxidative stress accelerates disc degeneration by influencing aging, inflammation, autophagy, and DNA methylation, and summarize some antioxidant therapeutic measures for IDD, indicating that antioxidant therapy for disc degeneration holds excellent promise.

## 1. Introduction

Intervertebral disc degeneration (IDD) was a common disabling condition that can impact the musculoskeletal system of the body [1]. In recent decades, lumbar disc degeneration, as a typical lesion of disc herniation, has been recognised as a main cause of disability worldwide, and 80% of adults are impacted at some point during their life [2]. The processes of degeneration included structural damage to the intervertebral disc and changes in cell number and various compositions. It is characterised by disc height reduction, annulus fibrosus (AF) tears, deprivation of proteoglycans (PGs), loss of water in the nucleus pulposus (NP), and calcification of cartilage endplate [3]. In addition, IDD was related to low back pain (LBP) [4]. According to statistics, the direct costs related to LBP were more than $90 billion every year in the United States alone [5]. The total socioeconomic impacts of LBP pose a significant challenge to society when considering the indirect costs of disability, including the patients' decreased productivity [6].

A comprehensive literature search was conducted in MEDILINE/PubMed, Embase, Chinese National Knowledge Infrastructure, Web of Science, Library and Information Science Abstracts, Scopus, and the National Library of Medicine catalog up until September 24, 2021. Reference lists were scrutinized, and we categorized them to complete this review.

Multiple factors contributed to IDD, consisting of degenerated NP and AF, DNA methylation, cellular senescence and oxidative stress, extracellular matrix (ECM), matrix metalloproteinases (MMPs), advanced glycation end products (AGEs), and reactive oxygen species (ROS) [7]. Among them, oxidative stress acts as a key role in the pathogenetic process of IDD, which has attracted more and more attention in recent years. In this review, we focus on the role of oxidative stress in the pathogenesis of disc degeneration as well as the various methods used to treat IDD by reducing oxidative stress.

## 2. Intervertebral Disc Degeneration

### 2.1. Normal Intervertebral Disc

**Figure 1:**
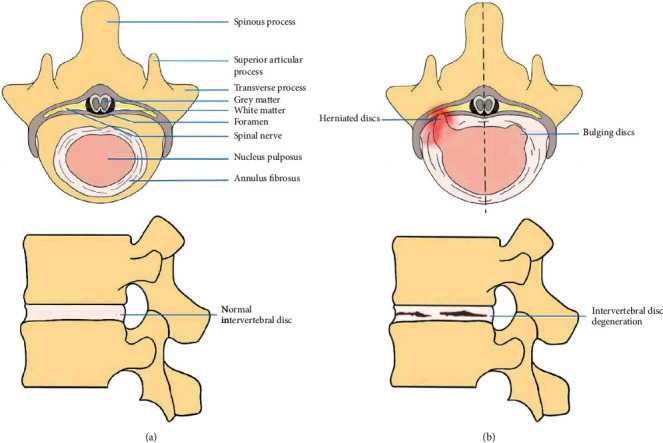
(a) Shows a normal disc with a horizontal view above and a sagittal view below. (b) Shows a disc pathology with a horizontal view of a herniated disc and bulging disc above and a sagittal view of disc degeneration below.

The IVD is a complex tissue, which consists of the NP, the AF, and the endochondral plate (EP), which is shown in Figure 1. NP is the colloidal central part of the IVD. It is made of water (70-90% of total weight), proteoglycans, and type II collagen. Type II collagen fibrils are disordered and diluted in the whole base network [8]. Nucleus pulposus cells were present in a colloidal matrix consisting of type II collagen and proteoglycans, which are essential to resisting axial compression forces and pressure on the spine [9]. The NP is surrounded by another IVD part, named fibrous ring, which can be further classified into its two subsections: outer AF (OAF) and inner AF (IAF) [10, 11]. The IAF is also called the “transition region” between NP and OAF because it has the features of both of NP and OAF regions [10]. The AF is a distinct structure consisting of 15-25 concentric circles (posterior vs. lateral) named lamella. Every lamella includes parallel collagen fibers (type I and II) arranged at an oblique angle with respect to the axis of compression [12, 13]. AF cells were present in a collagen-rich matrix and resist lateral expansion of the IVD while holding a weight-bearing position. EP cells are chondrocytes distributed in a hyaline cartilage matrix, which incorporates the intervertebral disc with the covering vertebrae [9]. The mechanical function of the intervertebral disc was determined by the extracellular matrix, which is mainly composed of two major macromolecules: collagen and aggrecan. Collagen offers tensile strength to the disc and connects the tissue to the bone. Aggrecan was in charge of the maintenance of tissue hydration through osmotic pressure regulation, which is the main proteoglycan of the disc [14].

Healthy intervertebral disc has an avascular and nonarterial structure that cannot transport nutrients through blood vessels, and its nutrients primarily diffuse through the end plate [15]. Its microenvironment was featured by hypoxia (1-2% oxygen), low nutrients, and an acidic pH caused by the accumulation of lactic acid accumulation [16]. When the cells die or become dysfunctional, in an acidic environment, IVD denaturation happens. During the degeneration process, the IVD becomes dehydrated and vascularised with nerve implantation [17]. Although it is not very common, the physiological alterations of the IVD were considered to precipitate or be related to various clinical symptoms or conditions, such as low back and/or lower extremity pain, numbness, spinal stenosis, and disc herniation [18]. The point is that despite being normally avascular, changes in tissue integration caused increased vascular and nerve growth in the disc, which could turn into a source of peripheral neuropathy, leading to pain, weakness, and numbness because of nerve damage [15].

### 2.2. Pathological Manifestations of the Intervertebral Disc

NP is the colloidal, greatly hydrated central area of the IVD that is encompassed by AF. EPs are located at the intersection of the IVD and vertebra, linking the two together [19, 20]. Due to the weak connection between the vertebral body and the IVD, the EP trabecular microinjury, by which abnormal pressure is transmitted by the neighboring NP and AF tissues directly, results in bulging or the herniation of the IVD [21, 22]. The NP area is mainly composed of notochordal cells, but with senility, chondrocyte-like cells gradually displace the original notochordal cells [20]. Chondrocyte-like cells have less capacity of keeping water than the resident notochordal cells; therefore, this age-associated change eventually leads to a decreased state of IVD hydration, and an inflexible functional spinal unit (FSU) [23, 24]. Moreover, fluid pressure and flow within the IVD change due to dehydration [25]. Cells inhabiting each region of the IVD evoke biochemical alterations (reduction of proteoglycans, glycosaminoglycans, aggrecan, and type II collagen as well as a promotion of type I collagen) through various molecules like cytokines, enzymes, and growth factors. When anabolic to catabolic transition of gene expression occurs in these cells, resulting in increased secretion of matrix-degrading enzymes, structural and mechanical changes begin [8]. At the molecular level, tumour necrosis factor alpha (TNF-*α*) and interleukin 1 (IL-1) are involved in the modulation of catabolic processes in degenerated intervertebral disc cells [26]. A few families of proteases participate in the breakdown of the ECM disc, including matrix metalloproteinases (MMPs), agglutinins, and cathepsins [27]. Among them, MMP-3 and MMP-13 were considered the critical factors in the ECM degradation in IVD [28, 29]. Being a crucial nutrient pathway for the IVD, calcified EP decreases the interchange of nutrients and metabolites in the IVD, while the reduction of nutrient and increased metabolite also exert key effects in suppressing the generation of ECM [30, 31]. In addition, the early period of IDD involves disrupting the balance between anabolic and catabolic activities (e.g., reducing collagen and glycosaminoglycans) and aberrant accumulation of proinflammatory factors, such as nitric oxide (NO), leukotrienes, and lactic acid [32, 33]. These pathophysiological change not only speed up disc degeneration but also serve as a trigger for pain. In the following, we will elucidate some of the mechanisms by which oxidative stress in turn causes changes in these cells or molecules, which then cause disc degeneration.

## 3. Oxidative Stress and IDD

### 3.1. Oxidative Stress

Oxidative stress is caused by the loss of balance between the generation of free radicals and reactive metabolites, which are called oxidants or ROS, and their removal by protective mechanisms known as antioxidants. The loss of balance resulted in damage to key biomolecules and cells, with underlying influence for the integral organism [34].

ROS are a series of erratic and evidently reactive molecules, which have free radicals or not, consisting of superoxide anions (O_2_^−^), hydroxyl radicals (OH^−^), hydrogen peroxide (H_2_O_2_), and hypochlorite ions (OCl^−^) [35]. Moreover, reactive nitrogen species (RNS), for example NO, are considered members of the ROS family because of their analogous effects to ROS, which are generated by the oxygen-using metabolic courses of cells [35]. That is to say, the generation of ROS is an unavoidable cost of aerobic metabolism [3]. RNS was able to further produce other reactive species by triggering excessive lipid peroxidation, for example, reactive aldehyde-malondialdehyde and 4-hydroxynonenal [36].

Highly reactive oxidative molecules always had deleterious influence on living cells. These molecules can be both free radicals and nonradicals (for instance, H_2_O_2_), but they have similar capacity to readily acquire electrons from molecules with which they interact, producing a series of reactions and eventually causing structural damage to the cell. Within these categories, the molecules produced by the ROS and RNS had the primary biological effect, as they are generated in vivo at the highest concentrations. Therefore, the nitrooxidative stress could also be classified as oxidative stress [37].

Oxidative stress occurred when the balance between generation and removal of free radicals or reactive metabolites is interrupted (e.g., antioxidant mechanisms in vivo are important to treat various diseases and maintain health.

As antioxidant enzymes, superoxide dismutase, catalase, and glutathione peroxidase (GSH-Px or GPx) are generally in charge of scavenging H_2_O_2_ and peroxides. They were distributed in various cellular parts and synergistically interact with other molecules, including catalase, thioredoxin (Trx), and glutathione, and low-molecular-weight antioxidants (e.g. GSH, tocopherol, and ascorbate) to adequately remove ROS [38–40]. And exogenous antioxidants formulate the crucial ROS detoxifying system, including reduced glutathione, carotenoids, and vitamins C and E [41, 42].

### 3.2. Oxidative Stress and IDD

Oxidative stress is involved in a variety of diseases, such as IDD, Alzheimer's, atherosclerosis, cancer, diabetes, and Parkinson's. We will focus on the correlation between oxidative stress and IDD.

Local oxidative stress is common in the intervertebral disc environment. In plasma from IDD patients or rats, superoxide dismutase activity significantly decreased and levels of various oxidative stress biomarkers sharply increased, consisting of phospholipase A, fructosamine, malondialdehyde, peroxidation potential, total hydrogen peroxide, advanced oxidation protein products, and NO [43, 44]. Furthermore, in degenerative discs, the balance between ROS generation and ROS elimination is impaired. Thus, oxidative stress is triggered in the degenerative disc microenvironment. Oxidative stress promoted the reduction in the amount of normal cells in the IVD microenvironment. In addition, the ROS can alter matrix proteins and trigger oxidative damage for the extracellular matrix of IVD, thus damaging the mechanical properties of IVDs. Moreover, the ROS could induce oxidative injury to DNA, lipids, and proteins. At the same time, metabolic waste of oxidative stress gradually increases in degenerated discs [3].

**Figure 2:**
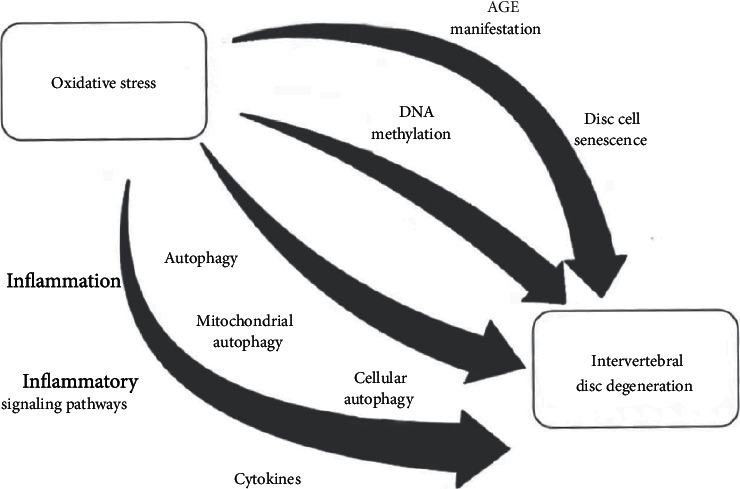
Oxidative stress affects disc degeneration through cellular senescence, inflammation, autophagy, and DNA methylation

In summary, both systemic and oxidative stress were aggravated in the course of IDD, indicating that oxidative stress plays a key role in the development progress of IDD [3], and a series of pathophysiological mechanisms were involved in this progress, including matrix metabolism, inflammation, apoptosis, DNA methylation, autophagy, and the senescence of disc cells, which is shown in Figure 2.

#### 3.2.1. Oxidative Stress and Disc Cell Senescence in IDD

The intervertebral discs experience age-related degenerative variations much earlier than other tissues [45]. An excess of mechanical load, diabetes, and chronic smoking, as well as acute disc damage, could aggravate the stresses induced by senescence. The senescence of disc cells not only aggravates the loss of disc cell viability and function due to numerous exhaustion but also releases matrix proteases, cytokines, and chemokines to impact the local microenvironment of IVD. And the avascularity of the IVD not only affected the immune clearance of the aged disc cells, thus inducing inflammatory reactions and catabolic metabolism, but also affected the disc microenvironment, accelerating IDD [46]. Actually, in addition to alterations in gene expression and metabolic control, the rate of aging was related to high levels of ROS and/or RNS generation [47].

As a member of the ROS family, the role of H_2_O_2_ in the intervertebral disc has been extensively studied. H_2_O_2_ rapidly promoted ROS production and DNA damage in NP cells of human [48]. H_2_O_2_ could activate various signalling pathways, such as p38 MAPKs, ERKs, and JNKs pathways, and trigger the nuclear translocation of NF-*κΒ* and Nrf2 [49]. Meanwhile, H_2_O_2_ could also trigger premature senescence of cartilage endplate cells via the p53-p21-Rb pathway [50]. Moreover, p53 was also deemed to be a sign of cellular aging [51]. The activation of the ATM-Chk2-p53-p21-Rb and p16-Rb signalling pathways triggered the premature senescence of NP cells in human [49, 52]. H_2_O_2_ could also cause senescent cells to present a catabolic phenotype, primarily characterised by an increase of extracellular matrix-degrading enzymes (MMP-1, MMP-2, MMP-9, and ADAMTS-5), by a decrease of their suppressant (TIMPs) and by several proteoglycans, such as aggrecan, which is a major component of the myeloid nucleus [49].

In addition, hyperglycaemia-induced excessive production of ROS also accelerated the aging of rat annulus fibrosus and notochordal cells via the p16-Rb pathway [53]. It is important to note that p16 has a significant role in the pathogenesis of IDD, and its loss attenuates IDD by facilitating the cell cycle and suppressing salicylazosulfapyridine, cellular aging, and oxidative stress [46]. Moreover, a number of studies have also confirmed that sirtuins were implicated in the onset and development of IDD. In particular, SIRT1, SIRT2, SIRT3, and SIRT6 have been shown to mediate the aging of IVD cells by being involved in the processes of inflammation, oxidative stress, and mitochondrial damage [54].

#### 3.2.2. Oxidative Stress and Advanced Glycation End Products (AGEs) in IDD

In the presence of oxidative stress, the increase of ROS led to the trigger of lipid peroxidation and glycosylation reactions, which result in the increased endogenous generation of reactive aldehydes and their by-products, including glyoxal, methylglyoxal, malondialdehyde, and 4-hydroxy-2-nonenal, leading to advanced lipid oxidation end products and advanced glycosylation end products (ALE and AGE, respectively) [55]. Interestingly, the increase of AGEs is the most compelling evidence of age-related oxidative damage to the disc, which accelerates the process of IVD degeneration via promoting apoptosis and hindering the metabolism of the extracellular matrix [56]. The most distinctive AGEs in cartilage and intervertebral discs were pentosidine and carboxymethyl lysine [57]. The former is distributed in collagen, which also crosslinks collagen molecules and may have a significant role in aggravated collagen stiffness and the dysfunction of cartilage biomechanics with age [58]. The latter is also present in collagen and has been found to accumulate in the proteins of intervertebral discs, with age as a marker of oxidative stress-related changes [49]. Immunomorphometric analysis showed higher levels of carboxymethyl lysine (CML; a biomarker of oxidative proteins) in the aged discs of elderly patients in comparison with the normal discs of younger patients, proving the existence of oxidative stress in IVDs [47].

AGEs were involved in the process of cartilage degeneration, resulting in age-dependent degenerative changes and covalent crosslinking of ECM adhesion proteins (e.g., collagen and laminin) [59, 60]. Moreover, AGEs accumulated in NP cells and subsequently triggered inflammatory responses in NP tissues and a degenerative phenotype via NLRP3 inflammasome, which was associated with AGE (RAGE)/NF-*κ*B pathway as well as for mitochondrial injury caused by mitochondrial reactive oxygen species production, calcium mobilisation, and mitochondrial permeability transition pore promotion [61]. In these pathways, both RAGE and mitochondrial dysfunction initiate NLRP3 and pro-IL-1*β* activation as stimulating signals of NF-*κ*B activity, indicating that AGEs enhance oxidative stress and IL-1*β* release results in the senescence of IVD [61].

Furthermore, Sirtuin3 (SIRT3) dysfunction and mitochondrial antioxidant networks were critical mechanisms of AGE-caused oxidative stress and apoptosis of NP cell in human. The activity of human NP cells was markedly inhibited by AGE intervention, primarily due to apoptosis. Moreover, activation of the mitochondrial apoptotic pathway was observed following AGE intervention. Furthermore, AGEs significantly exacerbated the production of mitochondrial ROS and prolonged promotion of the mitochondrial permeability transition pore, along with an increase in Bax protein levels and a reduction in Bcl-2 protein levels in mitochondria. These impacts could be alleviated by the antioxidants (2-[2, 2, 6, 6-tetramethylpiperidin-1-oxo-4-ylamino]-2-oxoethyl) triphenylphosphonium chloride and Visomitin [62].

#### 3.2.3. Oxidative Stress and Inflammation in IDD

Inflammation is a complicated immune response that is able to maintain tissue homeostasis during infection. A cascade of signals in response to pathogens, damaged cells, or stimuli led to the activation of immune cells. Due to the excessive production of proinflammatory cytokines and tissue damage, persistent and intense inflammation may result in severe disease [63].

Disc degeneration was regulated by aberrant generation of proinflammatory molecules released by NP and fibrocartilage annulus (fibrocartilage tissue containing NP) cells via macrophages, T cells, and neutrophils [64–66]. Thus, disc degeneration and inflammation were closely crosslinked. How does oxidative stress trigger an inflammatory response in the disc degeneration process? Normally, inflammatory tissues were connected with activated levels of reactive species (ROS and RNS) which were produced by immune cells and are crucial for protecting against foreign pathogens [67]. They are greatly active and easily react with biomolecules, such as lipids, proteins, and various metabolites. By promoting oxidation, the nitrosation and nitrification of a series of biomolecules dominated cellular signalling [68, 69]. These signalling cascades, known as “redox signalling,” strictly modulate the inflammatory response. Therefore, clarifying the intricate effect of ROS/RNS-caused redox signalling in inflammation would help to develop new treatment methods associated with inflammation-related disorders [67].

In addition, ROS/RNS could be reduced by various antioxidants, resulting in the alleviation of inflammation. It is clear that sustained excessive generation of ROS/RNS, caused by dysfunction of the cellular prooxidant-antioxidant system, will impair critical biomolecules and cells, resulting in excessive inflammatory reactions [70–72]. For instance, glutathione peroxidase-1 was an essential antioxidant enzyme, and its deficiency could initiate proinflammatory redox signalling [73, 74].

The ROS can play a role of signalling messenger in various signalling pathways, such as NF-*κ*B, mitogen-activated protein kinase (MAPK), and lipid signalling pathways (e.g., phospholipase, protein kinase C, and phosphatidylinositol-3 kinase (PI3K)/Akt) [41]. These pathways could mediate different cellular processes, including cell survival, proliferation, and inflammation. Among them, NF-*κ*B and MAPK signalling pathways play a key role in the course of inflammation.

NF-*κ*B was an indispensable molecule to modulate transcription of genes encoding proinflammatory cytokines, adhesion molecules, and chemokines as well as growth factors and inducible enzymes, which was a possible treatment target for inflammatory disease therapy [75]. H_2_O_2_ and peroxynitrite could modulate NF-*κ*B, which is involved in the metabolism and apoptotic rate of NP cells [33, 76].

Similar to p38 and JNK, extracellular regulated kinase 1/2 (ERK1/2) was a main type of MAPK kinase. ERK1/2 was essential for maintaining the normal physiological function of inflammatory cells. ROSs were able to deactivate the phosphatase in charge of dephosphorylating ERK1/2, leading to sustained activation of ERK1/2. The ROS-triggered activation of JNK and p38 was relied on the oxidation of thioredoxin (TRX) through multimerization of apoptosis signal-regulating kinase [77, 78].

In addition, RNS exerted significant proinflammatory effects on disc degeneration via inducing tissue damage, lipid peroxidation, and oxidative injury. Overload of NO induced S-nitrosylation and inhibition of NF-*κ*B and aggravates apoptotic rate of inflammatory cells, thus suppressing inflammation [79, 80].

Meanwhile, the ROS played an essential role in activating NLRP3 inflammasome, which was able to perceive pathogens and injuries, thus triggering the generation of inflammatory factors [81]. NLRP3 inflammasome also exerted harmful effects on metabolism and aggravates apoptosis in NP cells and leads to IVD degeneration [82]. Furthermore, mitochondrial dysfunction and mitochondrial ROS production promoted the activation of the NLRP3 inflammasome through various mechanisms. The NF-*κ*B signalling pathway participated in TNF-*α*-regulated NLRP3 inflammasome activation [83].

Meanwhile, ATP-triggered ROS accumulation could trigger the activation of Akt and ERK1/2 via glutathionylating PTEN, which is necessary for activating NLRP3 inflammasome and generating IL-1*β* and IL-18 in macrophages [84]. In addition, the TRX-interacting protein was a crucial regulator in the ROS-caused activation of the NLRP3 inflammasome [85].

**Figure 3:**
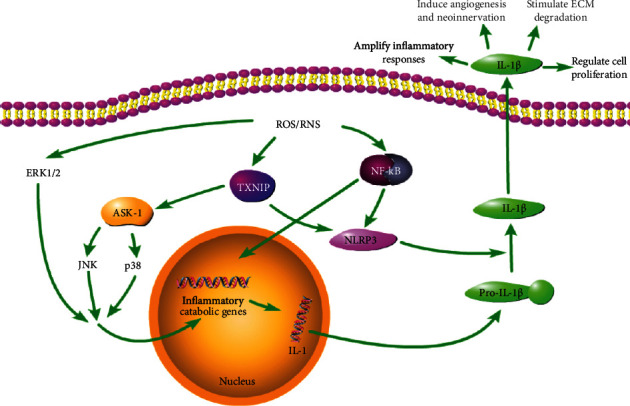
(1) NF-*κ*B signalling pathway. First, ROS/RNS regulates NF-*κ*B activity, including activating the NF-*κ*B signalling pathway and promoting inflammatory gene transcription. In addition, ROS/RNS plays an indispensable role in the activation of NLRP3 inflammasome and its oxidation of TRX causes TRX-interacting protein to bind to NLRP3, eventually producing IL-*β*. (2) MAPK signalling pathway. ROS/RNS triggers oxidation of TRX-interacting protein, leading to apoptosis signal-regulating kinase dissociation and activation of JNK and p38 pathways through multimerization of the apoptosis signal-regulating kinase, and activates the ERK1/2 pathway. Ultimately, ERK1/2, JNK, and p38 promote gene transcription, ultimately inducing IL-*β* production.

In fact, under certain circumstances, both ROS production and stimulation of antioxidants were necessary for activating inflammasome. IL-1*β* production, which was triggered by PAMP in monocytes of human, is modulated by biphasic oxidative events, including rapid oxidative stress and prolonged antioxidant responses because the NOX suppressor or TRX reductase could inhibit IL-1*β* release [86]. The abovementioned main process is briefly summarized in Figure 3.

#### 3.2.4. Oxidative Stress and Autophagy in IDD

Autophagy is a process of catabolism that recycles cellular components and waste organelles caused by various stress conditions, including nutrient insufficiency, viral infection, and genotoxic stress [37].

There are three major types of autophagy, all of which ultimately lead to lysosome-regulated degradation. The first is macroautophagy (hereafter called autophagy), which causes the creation of a double-membrane vesicle (autophagosome) to clear spoiled organelles and biomolecules. The second is microscopic autophagy, through which the cytosolic material is devoured by lysosomes directly, and the third is chaperonin-mediated autophagy. Autophagy was a very susceptible process, which is triggered by almost all the stressful factors that impact cellular homeostasis [87]. The accumulated evidence showed that oxidative stress is the point of confluence of these stimuli. The reactive ROS and RNS were the primary intracellular signal transducers to sustain autophagy [37]. In the rat model, nondegenerated rat NP and AF cells showed low levels of autophagy, whereas the autophagic activity was significantly higher in degenerative rat NP and AF cells [88, 89]. This suggests that autophagy is an important influencing factor in disc degeneration.

Autophagy and oxidative stress were correlated to each other in a more closely and coordinated way than via simple on/off signals. Several studies have shown that ROS can activate the MAPK pathway; for example, increasing ROS in plants activates the MAPK pathway [90], and excessive ROS production in Daphnia pulex induces activation of the downstream MAPK pathway [91], and similarly, ROS has been reported to activate the MAPK pathway in chicken cardiomyocytes [92]. A study on rats showed that the ROS could activate the MAPK pathway to trigger autophagy in NP cells of rat and to modulate the generation of TNF-*α*, matrix metalloproteinase-3 (MMP-3), cyclooxygenase-2, and aggrecan expression in AF cells of rat [93, 94]. Moreover, antioxidant treatment could suppress autophagy, indicating the key role of redox imbalance in advancing this process. The induction of autophagy by mitochondria after ROS generation provided a fast (on/off) response regulated by redox-sensitive proteins, in which the adenosine monophosphate activated protein kinase (AMPK) might be an important candidate.

AMPK could be activated during H_2_O_2_ exposure, specifically via the S-glutathionylation of reactive cysteines situated in the *α*-subunit and the *β*-subunit [95, 96]. Upon the deprivation of nutrients, cells actively squeezed out GSH via the drug efflux pump and the multidrug-resistant protein 1, thus enabling oxidation-sensitive proteins to be oxidatively modified [97]. Meanwhile, Cys81 (SH→Sox) of autophagy-related gene 4 was oxidised, which resulted in suppressing its “delipidation” activity on LC3 and leads to the increase of proautophagic LC3-II isoforms [98].

The overproduction of ROS enhanced the NP cell autophagy in rat via the AMPK/mTOR pathway [99]. In addition, H_2_O_2_ enhanced NP cell autophagy in rat via the ERK/mTOR pathway [93]. Mechanical stress-induced ROS hyperproduction was engaged in stress-caused autophagy in NP cells of rat. High glucose stress enhanced ROS generation and causes the upregulation of autophagy-related gene expression in notochord cells of rat [100].

Moreover, extensive alterations in the thiol redox status were implemented by the generation of decreased GSH to the extracellular environment by MRP1. In a redox-independent pattern, p62 could cause phosphorylation on Ser351 upon binding to the ubiquitinated protein aggregates, thus sequestering Keap1 and causing its separation from Nrf2. Thus, Nrf2 was no longer degraded via the ubiquitin-3 proteasome system; instead, it was transferred to the nucleus, where it combined to the antioxidant response elements situated in the promoter regions of antioxidant genes and initiates their transcription [101]. Notably, p62 included a zinc finger motif, which was rich with a variety of cysteine sequences that were indispensable for metal binding and that could be modulated by redox. Similar to other kinds of ZZ-containing proteins, p62 might undergo oxidative and structural changes that could change its effect in autophagy [102].

Furthermore, circular RNAs were a type of endogenous noncoding RNAs with a closed-loop structure that can serve as miRNA sponges, offering located areas for miRNAs to modulate the level of target genes [103]. There was growing evidence that some circular RNAs could act as miRNA suppressants and be related to the progression of IDD [104]. One type of circular RNAs, circERCC2, has been found to be suppressed in IDD and led to the progression of this disease. Meanwhile, Mir182-5p, recognised as a straightforward target of circErcc2, suppressed the expression level of SIRT1 (sirtuin1), thereby inhibiting mitosis. Thus, circErcc2 overexpression could markedly promote mitophagy by targeting Mir182-5p-SIRT1 to respond to oxidative stress [105].

In summary, there is a close relationship between oxidative stress and autophagy in the progression of disc degeneration.

#### 3.2.5. Oxidative Stress and DNA Methylation in IDD

A large number of mechanisms to modulate gene expression and cell fate have been gradually recognised, which were called epigenetics [106]. One of the most widely studied epigenetic regulation was DNA methylation [107], which induced gene silencing by suppressing the entry of transcriptional activators into the target binding site or by reactivating the structural domain of methyl-binding proteins [108]. DNA methylation could also evoke alterations in gene expression without altering the DNA sequence by creating 5-methylcytosine through the addition of methyl to cytosine in CpG-containing nucleotides [109]. DNA methylation usually serves to silence genes when methylation was stabilised in the promoter and enhancer regions of genes, while methylation, which was located in the gene body region, usually triggers an enhancement of gene expression [110].

DNA methylation was a crucial mechanism to facilitate certain gene expression for normal development, and abnormal epigenetic changes were recognised to play an important role in various diseases, including cancer and neurodegenerative disorders [111, 112]. In a recent comparative study on genome-wide DNA methylation profiles, remarkable distinctions of DNA methylation profiles between early and late phases of IVD degeneration in human were observed, suggesting a role for DNA methylation in the progression of IDD in human [113].

DNA methylation could be modulated differently in genes related to signalling pathways, including the NF-*κ*B, MAPK-ERK, and Wnt signalling pathways, which were situated upstream to the transcription of these catabolic molecule genes [113]. NF-*κ*B activation, which played a crucial part in inflammation via triggering the transcription of proinflammatory genes, has been proven to aggravate disc degeneration via promoting the release of matrix-degrading enzymes, including MMPs and ADAMTSs [45]. Three hypermethylated genes (CARD14, EFHD2, and RTKN2) have been identified in the late phase of disc degeneration, which was related to the modulation of the NF-*κ*B pathway. In addition, hypermethylated genes linked to the MAPK pathway, for instance, MAPKAPK5 and PRKCZ, have been recognised to have the capacity to modulate a variety of catabolic molecules [114–118].

ROS-dependent modifications were directly or indirectly associated with DNA methylation and demethylation. The 8-OHdG adduct disrupted DNA restriction nucleases and DNA methyltransferases, thereby changing the binding of transcription factors to DNA and leading to general DNA hypomethylation [119]. In addition, in vitro and in vivo studies have also shown that the ROS could trigger general genomic hypomethylation and DNA promoter hypomethylation through DNA methyltransferase upregulation and DNA methyltransferase complex production [120, 121].

In addition, the key proteins of the Wnt pathway were involved in the inflammatory reactions in the process of disc degeneration [17]. The Wnt signalling pathway regulated extracellular matrix metabolism via modulating proinflammatory stimulation. For instance, WNT5A, as a key member of the Wnt family, could be differentially methylated in the late stage of IVD degeneration [113].

In summary, there is a close relationship between oxidative stress and DNA methylation in the progression of disc degeneration.

## 4. Therapy

A deep understanding of the mechanisms through which disc degeneration occurs is essential for designing therapeutic strategies for therapy and the restoration of disc-related function. Because different therapeutic strategies may have different effects at different stages in the degenerative cascade, the selection of proper intervention is determined by the degree of disc degeneration. The IVD degenerative course is complex and multifactorial; therefore, solutions to improve this process are equally complex and may involve various solutions that rely on the disease phenotype and how it progresses [122].

Current treatments for disc degeneration include conservative and molecular therapies in the early stages and surgical procedures as the final treatment at the end of the disc degeneration process. Conservative therapy is divided into physical strengthening and physiotherapy, oral medications, and injections, while molecular therapy includes cellular therapy, growth factor therapy, and gene therapy.

Here, we summarize the antioxidants and other therapies that can alleviate disc degeneration by affecting oxidative stress.

### 4.1. Antioxidants

A series of antioxidants can exert therapeutic effects on degenerative disc cells and intervertebral disc degeneration, as is shown in Table 1.

#### 4.1.1. Polyphenols

Polyphenols belonged to natural compounds, which were found in vegetables, tea, wine, and chocolate; they have been widely studied for their antioxidant and anti-inflammatory characteristics [150, 151].

Resveratrol (RSV) is a polyphenolic compound found in various types of plants. The role of RSV in NP cells of human, rat, and bovine has been evaluated in previous studies. RSV inhibited NP cell death and enhanced the proliferation level of NP cell by stimulating silent information regulator 1 (SIRT1) and PI3K/Akt/caspase-3 pathways. In recent years, it has been reported that the combined application of 17*β*-estradiol and resveratrol alleviates apoptosis caused by IL-1*β* in NP cells of rat through PI3K/Akt/caspase-3 pathway [123, 124]. Among them, SIRT1 was a longevity gene, which can stimulate antioxidant expression and inhibits NF-*κ*B pathway activation in cells. SIRT1 activation has been demonstrated to diminish H_2_O_2_-caused senescence in human corneal epithelial cells in vitro [50]. Meanwhile, mitochondria were crucial targets of RSV, which regulates mitochondrial ROS generation and mitochondrial biogenesis through interacting with SIRT1 and energy metabolism through the transcriptional or enzymatic activation of SIRT3 [17, 152–154].

Moreover, RSV also inhibited the activation of a variety of transcription factors in NP cells. Consequently, PG synthesis in NP cells was promoted, and the release of matrix proteases and cytokines is suppressed in NP cells [125–128]. It is also worth noting that RSV could increase the production of aggrecan and SIRT1 and decrease the generation of MMP-3 and p16 to alleviate IDD [127].

Polyphenol epigallocatechin 3-gallate was considered a polyphenol redox cleaner, which could inhibit aging and apoptosis of NP cells following oxidative stress and suppress the generation of cytokines and MMPs by modulating the MAPK pathway and NF-*κ*B pathway in NP cell of human [52, 129].

Ferulic acid (FA, 4-hydroxy-3-methoxycinnamic acid) was a common type of phenolic antioxidant present in Chinese herbal medicine; it has anti-inflammatory, antiapoptotic, anticancer, and antiaging functions [155, 156]. FA could inhibit ROS accumulation in NP cells of rabbit, thereby delaying apoptosis. Moreover, FA could also induce the upregulation of aggrecan and type II collagen and the downregulation of MMP-3 production in NP cells of rabbit under oxidative stress conditions [130, 131].

Cordyceps sinensis is a common type of herbal medicine to treat chronic lung and kidney diseases in China. Cordycepin (3′-deoxyadenosine), as a type of the bioactive components, was extracted from Cordyceps sinensis. Lately, the anti-inflammation, antiaging, antioxidant, and anticancer functions of cordycepin have been reported [132, 133]. Cordycepin could also inhibit lipopolysaccharide- (LPS-) induced ROS generation and NF-*κ*B pathway activation to protect rat NP cells. Moreover, cordycepin could protect rat IVDs against LPS-caused degeneration at the level of ex vitro [134].

#### 4.1.2. ROS Scavengers

As an aromatic tricyclic o-quinone, pyrroloquinoline quinone (PQQ) had various health benefits, including growth-promoting capacity, antidiabetic activity, antioxidative effects, and neuroprotective abilities [157]. The antioxidant function of PQQ was executed through its role as an important cofactor for mitochondrial dehydrogenases and as a ROS sweeper [135, 136]. With respect to cells in the intervertebral disc, PQQ-inhibited H_2_O_2_ triggered the excessive production of ROS and then in protected NP cells of rat from H_2_O_2_-triggerred apoptosis in vitro. It also inhibited the H_2_O_2_-induced reduction of type II collagen and aggrecan in rat NP cells [137].

Fullerenes, as a specific type of carbon isotope, have been extensively researched for their unique material properties and potential technological applications, both in biology and medicine. Because of their continuous activity, distinctive nanostructure, and powerful cell membrane penetration ability, fullerenes were potent ROS scavengers [139, 140].

Fullerol, as a polyhydroxy derivant of fullerene, had a powerful scavenging capacity against ROS in comparison to superoxide dismutase and mannitol which was discovered in vitro to decrease ROS generation in NP cells of human and also impaired the promotion of matrix proteases and the reduction of type II collagen caused by H_2_O_2_ in NP cells of human. Furthermore, the intradiscal injection of fullerol exerted a protective effect on punctured rabbit intervertebral discs against degeneration by enhancing matrix synthesis and inhibiting ectopic ossification [141].

#### 4.1.3. Nonenzymatic Antioxidants

GSH was the one of the most abundant low-molecular-weight thiol compounds generated in cells. GSH played a key role in protecting cells against oxidative damage and the toxic effects of xenobiotic electrophiles as well as in maintaining redox homeostasis [143]. It could inhibit H_2_O_2_-induced apoptosis and matrix catabolic phenotypes and reduce IL-1*β*-caused ROS production in NP cells of human [142]. As a GSH precursor, N-acetylcysteine could reduce the level of ROS, thereby inhibiting the activation of the MAPK and AMPK/mTOR pathways in IVD cells of human and rat in vitro. The oral taking N-acetylcysteine suppressed oxidative stress, matrix catabolism, and inflammation in rat discs, which could alleviate IDD caused by needle piercing [49, 94].

#### 4.1.4. Oestrogen

Oestrogen is a type of steroid hormone that is primarily generated in the ovaries. Moreover, small amounts of oestrogen were also generated in the adrenal glands, male testes, and adipose tissue [144]. Women had three types of oestrogen: estrone, estradiol, and estriol. 17 beta estradiol was the main and effective form of oestrogen in the blood circulation of women [158].

Oestrogen has been demonstrated to exert significant modulatory effects on various human systems. This type of steroid hormones was initially released by the ovaries, which also has a regulatory role in the metabolism of the skeletal system [145–147]. In addition, oestrogen regulates IVD metabolism and strengthens the function of antioxidants; serum oestrogen levels were also inversely related to redox homeostasis [159].

The level of oestrogen had a significant impact on IVDs, and oestrogen deficiency can significantly exacerbate IDD, which is evidenced by the fact that the disc lesions and calcifications are becoming more serious [160]. Moreover, oestrogen deficiency could also result in more loss of IVD height and ECM as well as abnormalities in MMPs (MMP-3 and MMP-13) [148]. Meanwhile, oestrogen supplementation repaired pathological lesions and endplate calcification, promotes ECM expression, and then recovers IVD hydrophilicity and inhibited MMP-3 and MMP-13 expression. In addition, 17*β*-estradiol also counteracted against IL-1*β*-induced apoptosis in myeloid cells through decreasing MMP-3 and MMP-13 by the mitochondrial pathway [148]. Oestrogen could also reduce IVD cell apoptosis via various mechanisms, such as the suppression of the inflammatory cytokines IL-1*β* and TNF-*α*, catabolism reduction due to the suppression of matrix metalloproteinases, the promotion of integrin *α*2*β*1 and IVD anabolism, the upregulation of the PI3K/Akt pathway, and the reduction of oxidative damage [138, 149].

### 4.2. Others

#### 4.2.1. Mesenchymal Stem Cell-Derived Exosomes

Exosome is a type of extracellular vesicle, and the diameter range is 50-100 nm; they are secreted by nearly all types of cells. Exosomes included cytokines, proteins, lipids, and noncoding RNAs. Moreover, they are participated in cell-to-cell interactions by transferring their contents among various cells. Thus, exosomes have become essential mediators in the treatment of different diseases [161, 162]. Exosomes could exert anti-inflammatory effects in damaged NP cells via inhibiting the activation of inflammatory regulators and NLRP3 inflammasome. In addition, the impaired mitochondria can be recovered with mitochondrial proteins which were provided by exosomes in NP cells. They also contained antioxidant proteins, based on a proteomics database [163, 164]. Exosomes could also lower ROS generation in NP cell and reduce cellular apoptosis. In addition, a recent study firstly reported that mesenchymal stem cell-exosome therapy could suppress pyroptosis and could be a possible therapeutic method to treat IDD [165, 166].

#### 4.2.2. Growth Factors

Growth factor therapy consists of injecting biological factors directly into the IVD to increase synthesis of the extracellular matrix, slow degeneration, and block inflammation. Growth factors were peptides that act against receptors to elicit cellular effects, including proliferation, differentiation, apoptosis, and protein synthesis [167]. For example, bone morphogenetic protein-7 suppressed the proapoptotic role of H_2_O_2_ in vitro and sustains the capacity of matrix synthesis following oxidative stress. Insulin-like growth factor-1 improved the H_2_O_2_-induced premature aging of human AF cells in vitro [168, 169]. The hepatocyte growth factor protected NP cells against apoptosis caused by H_2_O_2_ in vitro, which reduced the generation of matrix proteases and proinflammatory cytokines in NP rabbit cells [170].

## 5. Future Outlooks and Conclusion

IDD is a significant risk factor for low back pain and disability, and the prevalence will continue to rise as the population continues to age. Therefore, to clarify the accurate mechanisms and find a safe and effective treatment for IDD is becoming more and more important. Although the specific mechanisms of IDD were not yet fully understood, what has come to be known is that IDD is a systemic degenerative process associated with multiple factors, among which the contribution of oxidative stress to IDD pathogenesis is complex and substantial, opening a novel horizon with respect to the pathogenetic mechanism of IDD.

In this review, we mainly summarize the relationships between oxidative stress and IDD, including aging, inflammation, autophagy, and DNA methylation. Moreover, antioxidant therapies are considered promising therapeutics for IDD, which are also summarized in the present review. In addition, we have summarized the abbreviations and their full names as follows, as is shown in Abbreviations. Looking forward, basic research experiments and clinical trials are still needed to develop novel and reliable therapeutic targets to treat IDD.

## Figures and Tables

**Table 1:** Therapeutic effects of antioxidants on degenerative disc cells and intervertebral disc degeneration.

Antioxidant		Therapeutic effects
Polyphenols	Resveratrol (RSV)	Inhibition of NP cell death and senescence [123, 124] Promoted NP cell proliferation [120, 123] Downregulates matrix protease and cytokine expression [125–128] Enhanced the synthesis of PG in NP cells ([125], [128], [126], [127])
	Polyphenol epigallocatechin 3-gallate	Inhibited NP cell senescence and apoptosis [52, 129] Inhibited the expression of cytokines and MMPs in NP cells ([52], [129])
	Ferulic acid (FA)	Inhibited the accumulation of ROS in NP cells [130, 131] Upregulated the expression of aggrecan and type II collagen [130, 131] Downregulated the expression of MMP-3 ([130], [131])
	Cordyceps militaris	Inhibition of ROS production [132, 133] Inhibition of NF-*κ*B pathway activation [134]
ROS scavengers	Pyrroloquinoline quinone (PQQ)	An important cofactor for mitochondrial dehydrogenase redox ROS scavenger [135, 136] Inhibited NP cell apoptosis [137] Inhibited downregulation of type II collagen and aggrecan in NP cells [138]
	Fullerenes	Powerful ROS scavenger ([139], [140])
	Fullerol	Reduction of ROS production in NP cells [141] Attenuated upregulation of matrix proteases and downregulation of type II collagen [142] Promoted matrix synthesis [141] Inhibited ectopic ossification [142]
Nonenzymatic antioxidants	Glutathione (GSH)	Inhibited NP cell apoptosis and stromal breakdown [141, 143] Reduced OS production ([49], [94])
	N-Acetylcysteine (NAC)	Reduced ROS levels [49, 94] Inhibited the activation of inflammatory signalling pathways [49, 94] Inhibited catabolism of intervertebral disc cells [49, 94] Inhibited autophagy and apoptosis of intervertebral disc cells [49, 94] Improved premature aging ([49], [94])
Oestrogen	Oestrogen	Enhanced antioxidant capacity [144] Upregulation of aggrecan and type II collagen expression [145–147] Downregulates the expression of MMP-3 and MMP-13 [148] Reduces oxidative damage and promotes autophagy ([149], [137])

## Abbreviations

AF: Annulus fibrosus
AGE: Advanced glycosylation end products
ALE: Advanced lipid oxidation end products
AMPK: Adenosine monophosphate activated protein kinase
ECM: Extracellular matrix
EP: Endochondral plate
ERK1/2: Extracellular regulated kinase 1/2
FA: Ferulic acid
GSH: Glutathione
H_2_O_2_: Hydrogen peroxide
IDD: Intervertebral disc degeneration
IL-1: Interleukin 1
LBP: Low back pain
MAPKs: Mitogen-activated protein kinases
MMP-3: Matrix metalloproteinase-3
MMPs: Matrix metalloproteinases
NO: Nitric oxide
NP: Nucleus pulposus
O_2_^−^: Superoxide anions
OCl^−^: Hypochlorite ions
OH^−^: Hydroxyl radicals
PI3K/Akt: Phosphatidylinositol-3 kinase
PQQ: Pyrroloquinoline quinone
RNS: Reactive nitrogen species
ROS: Reactive oxygen species
RSV: Resveratrol
SIRT1: Silent information regulator 1
SIRT3: Silent information regulator 3
TNF-*α*: Tumour necrosis factor alpha
Trx: Thioredoxin.

## References

[1] Colombini A., Lombardi G., Corsi M. M., Banfi G. (2008). Pathophysiology of the human intervertebral disc. *The International Journal of Biochemistry & Cell Biology*.

[2] Chenot J. F., Greitemann B., Kladny B., Petzke F., Pfingsten M., Schorr S. G. (2017). Non-specific low back pain. *Deutsches Ärzteblatt International*.

[3] Feng C., Yang M., Lan M. (2017). ROS: crucial intermediators in the pathogenesis of intervertebral disc degeneration. *Oxidative Medicine and Cellular Longevity*.

[4] Livshits G., Popham M., Malkin I. (2011). Lumbar disc degeneration and genetic factors are the main risk factors for low back pain in women: the UK Twin Spine Study. *Annals of the Rheumatic Diseases*.

[5] Dieleman J. L., Baral R., Birger M. (2016). US spending on personal health care and public health, 1996-2013. *JAMA*.

[6] Katz J. N. (2006). Lumbar disc disorders and low-back pain: socioeconomic factors and consequences. *The Journal of Bone and Joint Surgery. American Volume*.

[7] Rider S. M., Mizuno S., Kang J. D. (2019). Molecular mechanisms of intervertebral disc degeneration. *Spine surgery and related research*.

[8] Molladavoodi S., McMorran J., Gregory D. (2020). Mechanobiology of annulus fibrosus and nucleus pulposus cells in intervertebral discs. *Cell and Tissue Research*.

[9] Hayes A. J., Benjamin M., Ralphs J. R. (2001). Extracellular matrix in development of the intervertebral disc. *Matrix Biology*.

[10] Erwin W. M., Hood K. E. (2014). The cellular and molecular biology of the intervertebral disc: a clinician's primer. *The Journal of the Canadian Chiropractic Association*.

[11] Hsieh A. H., Twomey J. D. (2010). Cellular mechanobiology of the intervertebral disc: new directions and approaches. *Journal of Biomechanics*.

[12] Sakai D., Grad S. (2015). Advancing the cellular and molecular therapy for intervertebral disc disease. *Advanced Drug Delivery Reviews*.

[13] Smith L. J., Elliott D. M. (2011). Formation of lamellar cross bridges in the annulus fibrosus of the intervertebral disc is a consequence of vascular regression. *Matrix Biology*.

[14] Kos N., Gradisnik L., Velnar T. (2019). A brief review of the degenerative intervertebral disc disease. *Medieval Archaeology*.

[15] Hughes S. P., Freemont A. J., Hukins D. W., McGregor A. H., Roberts S. (2012). The pathogenesis of degeneration of the intervertebral disc and emerging therapies in the management of back pain. *Journal of Bone and Joint Surgery. British Volume (London)*.

[16] Huang Y. C., Leung V. Y., Lu W. W., Luk K. D. (2013). The effects of microenvironment in mesenchymal stem cell-based regeneration of intervertebral disc. *The Spine Journal*.

[17] Chen C., Jiang X., Zhao W., Zhang Z. (2013). Dual role of resveratrol in modulation of genotoxicity induced by sodium arsenite via oxidative stress and apoptosis. *Food and Chemical Toxicology*.

[18] Chen K., Wu D., Zhu X. (2013). Gene expression profile analysis of human intervertebral disc degeneration. *Genetics and Molecular Biology*.

[19] Hunter C. J., Matyas J. R., Duncan N. A. (2003). The three-dimensional architecture of the notochordal nucleus pulposus: novel observations on cell structures in the canine intervertebral disc. *Journal of Anatomy*.

[20] Hunter C. J., Matyas J. R., Duncan N. A. (2004). Cytomorphology of notochordal and chondrocytic cells from the nucleus pulposus: a species comparison. *Journal of Anatomy*.

[21] Adams M. A., Freeman B. J., Morrison H. P., Nelson I. W., Dolan P. (2000). Mechanical initiation of intervertebral disc degeneration. *Spine (Phila Pa 1976)*.

[22] Vernon-Roberts B., Pirie C. J. (1973). Healing trabecular microfractures in the bodies of lumbar vertebrae. *Annals of the Rheumatic Diseases*.

[23] Weiler C., Nerlich A. G., Schaaf R., Bachmeier B. E., Wuertz K., Boos N. (2010). Immunohistochemical identification of notochordal markers in cells in the aging human lumbar intervertebral disc. *European Spine Journal*.

[24] Zirbel S. A., Stolworthy D. K., Howell L. L., Bowden A. E. (2013). Intervertebral disc degeneration alters lumbar spine segmental stiffness in all modes of loading under a compressive follower load. *The Spine Journal*.

[25] Chuang S. Y., Popovich J. M., Lin L. C., Hedman T. P. (2010). The effects of exogenous crosslinking on hydration and fluid flow in the intervertebral disc subjected to compressive creep loading and unloading. *Spine (Phila Pa 1976)*.

[26] Ahn S. H., Cho Y. W., Ahn M. W., Jang S. H., Sohn Y. K., Kim H. S. (2002). mRNA expression of cytokines and chemokines in herniated lumbar intervertebral discs. *Spine (Phila Pa 1976)*.

[27] Wang Y. T., Wu X. T., Wang F. (2010). Regeneration potential and mechanism of bone marrow mesenchymal stem cell transplantation for treating intervertebral disc degeneration. *Journal of Orthopaedic Science*.

[28] Mavrogonatou E., Angelopoulou M. T., Kletsas D. (2014). The catabolic effect of TNF*α* on bovine nucleus pulposus intervertebral disc cells and the restraining role of glucosamine sulfate in the TNF*α*-mediated up-regulation of MMP-3. *Journal of Orthopaedic Research*.

[29] Yao Z., Nie L., Zhao Y. (2016). Salubrinal suppresses IL-17-induced upregulation of MMP-13 and extracellular matrix degradation through the NF-kB pathway in human nucleus pulposus cells. *Inflammation*.

[30] Rajasekaran S., Babu J. N., Arun R., Armstrong B. R., Shetty A. P., Murugan S. (2004). ISSLS prize winner: a study of diffusion in human lumbar discs: a serial magnetic resonance imaging study documenting the influence of the endplate on diffusion in normal and degenerate discs. *Spine (Phila Pa 1976)*.

[31] Townsend D. M., Tew K. D., Tapiero H. (2003). The importance of glutathione in human disease. *Biomedicine & Pharmacotherapy*.

[32] Fujii K., Yamazaki M., Kang J. D. (2019). Discogenic back pain: literature review of definition, diagnosis, and treatment. *JBMR Plus*.

[33] Risbud M. V., Shapiro I. M. (2014). Role of cytokines in intervertebral disc degeneration: pain and disc content. *Nature Reviews Rheumatology*.

[34] Ďuračková Z. (2010). Some current insights into oxidative stress. *Physiological Research*.

[35] Kim K. A., Yim J. E. (2015). Antioxidative activity of onion peel extract in obese women: a randomized, double-blind, placebo controlled study. *Journal of cancer prevention*.

[36] Reuter S., Gupta S. C., Chaturvedi M. M., Aggarwal B. B. (2010). Oxidative stress, inflammation, and cancer: how are they linked?. *Free Radical Biology & Medicine*.

[37] Filomeni G., De Zio D., Cecconi F. (2015). Oxidative stress and autophagy: the clash between damage and metabolic needs. *Cell Death and Differentiation*.

[38] Bindoli A., Fukuto J. M., Forman H. J. (2008). Thiol chemistry in peroxidase catalysis and redox signaling. *Antioxidants & Redox Signaling*.

[39] Filomeni G., Rotilio G., Ciriolo M. R. (2002). Cell signalling and the glutathione redox system. *Biochemical Pharmacology*.

[40] Flohé L. (2010). Changing paradigms in thiology from antioxidant defense toward redox regulation. *Methods in Enzymology*.

[41] Davalli P., Mitic T., Caporali A., Lauriola A., D'Arca D. (2016). ROS, cell senescence, and novel molecular mechanisms in aging and age-related diseases. *Oxidative Medicine and Cellular Longevity*.

[42] Pompella A., Corti A. (2015). Editorial: the changing faces of glutathione, a cellular protagonist. *Frontiers in Pharmacology*.

[43] Hou G., Lu H., Chen M., Yao H., Zhao H. (2014). Oxidative stress participates in age-related changes in rat lumbar intervertebral discs. *Archives of Gerontology and Geriatrics*.

[44] León Fernández O. S., Pantoja M., Díaz Soto M. T. (2012). Ozone oxidative post-conditioning reduces oxidative protein damage in patients with disc hernia. *Neurological Research*.

[45] Nerlich A. G., Bachmeier B. E., Schleicher E., Rohrbach H., Paesold G., Boos N. (2007). Immunomorphological analysis of RAGE receptor expression and NF-kappaB activation in tissue samples from normal and degenerated intervertebral discs of various ages. *Annals of the New York Academy of Sciences*.

[46] Che H., Li J., Li Y. (2020). p16 deficiency attenuates intervertebral disc degeneration by adjusting oxidative stress and nucleus pulposus cell cycle. *eLife*.

[47] Vo N., Niedernhofer L. J., Nasto L. A. (2013). An overview of underlying causes and animal models for the study of age-related degenerative disorders of the spine and synovial joints. *Journal of Orthopaedic Research*.

[48] Kim K. W., Chung H. N., Ha K. Y., Lee J. S., Kim Y. Y. (2009). Senescence mechanisms of nucleus pulposus chondrocytes in human intervertebral discs. *The Spine Journal*.

[49] Dimozi A., Mavrogonatou E., Sklirou A., Kletsas D. (2015). Oxidative stress inhibits the proliferation, induces premature senescence and promotes a catabolic phenotype in human nucleus pulposus intervertebral disc cells. *European Cells & Materials*.

[50] Zhou N., Lin X., Dong W. (2016). SIRT1 alleviates senescence of degenerative human intervertebral disc cartilage endo-plate cells via the p 53/p21 pathway. *Scientific Reports*.

[51] Xu J., Li H., Yang K. (2019). Hyper-osmolarity environment-induced oxidative stress injury promotes nucleus pulposus cell senescence in vitro. *Bioscience Reports*.

[52] Krupkova O., Handa J., Hlavna M. (2016). The natural polyphenol epigallocatechin gallate protects intervertebral disc cells from oxidative stress. *Oxidative Medicine and Cellular Longevity*.

[53] Park J. S., Park J. B., Park I. J., Park E. Y. (2014). Accelerated premature stress-induced senescence of young annulus fibrosus cells of rats by high glucose-induced oxidative stress. *International Orthopaedics*.

[54] Yi X., Guo W., Shi Q. (2019). SIRT3-dependent mitochondrial dynamics remodeling contributes to oxidative stress-induced melanocyte degeneration in vitiligo. *Theranostics*.

[55] Vistoli G., De Maddis D., Cipak A., Zarkovic N., Carini M., Aldini G. (2013). Advanced glycoxidation and lipoxidation end products (AGEs and ALEs): an overview of their mechanisms of formation. *Free Radical Research*.

[56] Sivan S. S., Tsitron E., Wachtel E. (2006). Age-related accumulation of pentosidine in aggrecan and collagen from normal and degenerate human intervertebral discs. *The Biochemical Journal*.

[57] Bank R. A., Bayliss M. T., Lafeber F. P., Maroudas A., Tekoppele J. M. (1998). Ageing and zonal variation in post-translational modification of collagen in normal human articular cartilage. The age-related increase in non-enzymatic glycation affects biomechanical properties of cartilage. *The Biochemical Journal*.

[58] DeGroot J., Verzijl N., Bank R. A., Lafeber F. P., Bijlsma J. W., TeKoppele J. M. (1999). Age-related decrease in proteoglycan synthesis of human articular chondrocytes: the role of nonenzymatic glycation. *Arthritis and Rheumatism*.

[59] Furber J. D. (2006). Extracellular glycation crosslinks: prospects for removal. *Rejuvenation Research*.

[60] Viguet-Carrin S., Roux J. P., Arlot M. E. (2006). Contribution of the advanced glycation end product pentosidine and of maturation of type I collagen to compressive biomechanical properties of human lumbar vertebrae. *Bone*.

[61] Song Y., Wang Y., Zhang Y. (2017). Advanced glycation end products regulate anabolic and catabolic activities via NLRP3-inflammasome activation in human nucleus pulposus cells. *Journal of Cellular and Molecular Medicine*.

[62] Song Y., Li S., Geng W. (2018). Sirtuin 3-dependent mitochondrial redox homeostasis protects against AGEs-induced intervertebral disc degeneration. *Redox Biology*.

[63] Žerovnik E., Ventura S., Kopitar-Jerala N. (2020). Special issue: "Inflammation, oxidative stress and protein aggregation; any links?". *Cell*.

[64] Kepler C. K., Markova D. Z., Hilibrand A. S. (2013). Substance P stimulates production of inflammatory cytokines in human disc cells. *Spine (Phila Pa 1976)*.

[65] Rand N., Reichert F., Floman Y., Rotshenker S. (1997). Murine nucleus pulposus-derived cells secrete interleukins-1-beta, -6, and -10 and granulocyte-macrophage colony-stimulating factor in cell culture. *Spine (Phila Pa 1976)*.

[66] Yamamoto J., Maeno K., Takada T. (2013). Fas ligand plays an important role for the production of pro-inflammatory cytokines in intervertebral disc nucleus pulposus cells. *Journal of Orthopaedic Research*.

[67] Lei Y., Wang K., Deng L., Chen Y., Nice E. C., Huang C. (2015). Redox regulation of inflammation: old elements, a new story. *Medicinal Research Reviews*.

[68] Chiurchiù V., Maccarrone M. (2011). Chronic inflammatory disorders and their redox control: from molecular mechanisms to therapeutic opportunities. *Antioxidants & Redox Signaling*.

[69] Miki H., Funato Y. (2012). Regulation of intracellular signalling through cysteine oxidation by reactive oxygen species. *Journal of Biochemistry*.

[70] Azad N., Rojanasakul Y., Vallyathan V. (2008). Inflammation and lung cancer: roles of reactive oxygen/nitrogen species. *Journal of Toxicology and Environmental Health. Part B, Critical Reviews*.

[71] Bryan N., Ahswin H., Smart N., Bayon Y., Wohlert S., Hunt J. A. (2012). Reactive oxygen species (ROS)--a family of fate deciding molecules pivotal in constructive inflammation and wound healing. *European Cells & Materials*.

[72] Gill R., Tsung A., Billiar T. (2010). Linking oxidative stress to inflammation: Toll-like receptors. *Free Radical Biology & Medicine*.

[73] Lubos E., Kelly N. J., Oldebeken S. R. (2011). Glutathione peroxidase-1 deficiency augments proinflammatory cytokine-induced redox signaling and human endothelial cell activation. *The Journal of Biological Chemistry*.

[74] Oelze M., Kröller-Schön S., Steven S. (2014). Glutathione peroxidase-1 deficiency potentiates dysregulatory modifications of endothelial nitric oxide synthase and vascular dysfunction in aging. *Hypertension*.

[75] Wang S., Liu Z., Wang L., Zhang X. (2009). NF-kappaB signaling pathway, inflammation and colorectal cancer. *Cellular & Molecular Immunology*.

[76] Loukili N., Rosenblatt-Velin N., Rolli J. (2010). Oxidants positively or negatively regulate nuclear factor kappaB in a context-dependent manner. *The Journal of Biological Chemistry*.

[77] Matsuzawa A., Ichijo H. (2008). Redox control of cell fate by MAP kinase: physiological roles of ASK1-MAP kinase pathway in stress signaling. *Biochimica et Biophysica Acta*.

[78] Son Y., Cheong Y. K., Kim N. H., Chung H. T., Kang D. G., Pae H. O. (2011). Mitogen-activated protein kinases and reactive oxygen species: how can ROS activate MAPK pathways?. *Journal of Signal Transduction*.

[79] Andreadis A. A., Hazen S. L., Comhair S. A., Erzurum S. C. (2003). Oxidative and nitrosative events in asthma. *Free Radical Biology & Medicine*.

[80] Sugiura H., Ichinose M. (2011). Nitrative stress in inflammatory lung diseases. *Nitric Oxide*.

[81] Osawa R., Williams K. L., Singh N. (2011). The inflammasome regulatory pathway and infections: role in pathophysiology and clinical implications. *The Journal of Infection*.

[82] Tang P., Gu J. M., Xie Z. A. (2018). Honokiol alleviates the degeneration of intervertebral disc via suppressing the activation of TXNIP-NLRP3 inflammasome signal pathway. *Free Radical Biology & Medicine*.

[83] Zhu H., Sun B., Shen Q. (2019). TNF-*α* induces apoptosis of human nucleus pulposus cells via activating the TRIM14/NF-*κ*B signalling pathway. *Artificial cells, nanomedicine, and biotechnology*.

[84] Cruz C. M., Rinna A., Forman H. J., Ventura A. L., Persechini P. M., Ojcius D. M. (2007). ATP activates a reactive oxygen species-dependent oxidative stress response and secretion of proinflammatory cytokines in macrophages. *The Journal of Biological Chemistry*.

[85] Zhou R., Tardivel A., Thorens B., Choi I., Tschopp J. (2010). Thioredoxin-interacting protein links oxidative stress to inflammasome activation. *Nature Immunology*.

[86] Tassi S., Carta S., Vené R., Delfino L., Ciriolo M. R., Rubartelli A. (2009). Pathogen-induced interleukin-1beta processing and secretion is regulated by a biphasic redox response. *Journal of Immunology*.

[87] Kroemer G., Mariño G., Levine B. (2010). Autophagy and the integrated stress response. *Molecular Cell*.

[88] Jiang L., Zhang X., Zheng X. (2013). Apoptosis, senescence, and autophagy in rat nucleus pulposus cells: Implications for diabetic intervertebral disc degeneration. *Journal of Orthopaedic Research*.

[89] Ye W., Zhu W., Xu K. (2013). Increased macroautophagy in the pathological process of intervertebral disc degeneration in rats. *Connective Tissue Research*.

[90] Jalmi S. K., Sinha A. K. (2015). ROS mediated MAPK signaling in abiotic and biotic stress- striking similarities and differences. *Frontiers in Plant Science*.

[91] Liu Z., Huang Y., Jiao Y. (2020). Polystyrene nanoplastic induces ROS production and affects the MAPK-HIF-1/NFkB-mediated antioxidant system in Daphnia pulex. *Aquatic Toxicology*.

[92] Cai J., Yang J., Liu Q. (2019). Mir-215-5p induces autophagy by targeting PI3K and activating ROS-mediated MAPK pathways in cardiomyocytes of chicken. *Journal of Inorganic Biochemistry*.

[93] Chen J. W., Ni B. B., Li B., Yang Y. H., Jiang S. D., Jiang L. S. (2014). The responses of autophagy and apoptosis to oxidative stress in nucleus pulposus cells: implications for disc degeneration. *Cellular Physiology and Biochemistry*.

[94] Suzuki S., Fujita N., Hosogane N. (2015). Excessive reactive oxygen species are therapeutic targets for intervertebral disc degeneration. *Arthritis Research & Therapy*.

[95] Cardaci S., Filomeni G., Ciriolo M. R. (2012). Redox implications of AMPK-mediated signal transduction beyond energetic clues. *Journal of Cell Science*.

[96] Zmijewski J. W., Banerjee S., Bae H., Friggeri A., Lazarowski E. R., Abraham E. (2010). Exposure to hydrogen peroxide induces oxidation and activation of AMP-activated protein kinase. *The Journal of Biological Chemistry*.

[97] Desideri E., Filomeni G., Ciriolo M. R. (2012). Glutathione participates in the modulation of starvation-induced autophagy in carcinoma cells. *Autophagy*.

[98] Kongara S., Karantza V. (2012). The interplay between autophagy and ROS in tumorigenesis. *Frontiers in Oncology*.

[99] Chen J. W., Ni B. B., Zheng X. F., Li B., Jiang S. D., Jiang L. S. (2015). Hypoxia facilitates the survival of nucleus pulposus cells in serum deprivation by down-regulating excessive autophagy through restricting ROS generation. *The International Journal of Biochemistry & Cell Biology*.

[100] Park E. Y., Park J. B. (2013). High glucose-induced oxidative stress promotes autophagy through mitochondrial damage in rat notochordal cells. *International Orthopaedics*.

[101] Xiao B., Hong L., Cai X., Mei S., Zhang P., Shao L. (2019). The true colors of autophagy in doxorubicin-induced cardiotoxicity. *Oncology Letters*.

[102] Giles N. M., Gutowski N. J., Giles G. I., Jacob C. (2003). Redox catalysts as sensitisers towards oxidative stress. *FEBS Letters*.

[103] Kristensen L. S., Andersen M. S., Stagsted L. V. W., Ebbesen K. K., Hansen T. B., Kjems J. (2019). The biogenesis, biology and characterization of circular RNAs. *Nature Reviews. Genetics*.

[104] Li Y., Wang X., Xu H. (2021). Circ_0040039 may aggravate intervertebral disk degeneration by regulating the miR-874-3p-ESR1 pathway. *Frontiers in Genetics*.

[105] Xie L., Huang W., Fang Z. (2019). CircERCC2 ameliorated intervertebral disc degeneration by regulating mitophagy and apoptosis through miR-182-5p/SIRT1 axis. *Cell Death & Disease*.

[106] Goldberg A. D., Allis C. D., Bernstein E. (2007). Epigenetics: a landscape takes shape. *Cell*.

[107] Jaenisch R., Bird A. (2003). Epigenetic regulation of gene expression: how the genome integrates intrinsic and environmental signals. *Nature Genetics*.

[108] Moore L. D., Le T., Fan G. (2013). DNA methylation and its basic function. *Neuropsychopharmacology*.

[109] Dor Y., Cedar H. (2018). Principles of DNA methylation and their implications for biology and medicine. *Lancet*.

[110] Jones P. A. (2012). Functions of DNA methylation: islands, start sites, gene bodies and beyond. *Nature Reviews. Genetics*.

[111] Jin Z., Liu Y. (2018). DNA methylation in human diseases. *Genes & diseases*.

[112] Sandoval J., Esteller M. (2012). Cancer epigenomics: beyond genomics. *Current Opinion in Genetics & Development*.

[113] Ikuno A., Akeda K., Takebayashi S. I., Shimaoka M., Okumura K., Sudo A. (2019). Genome-wide analysis of DNA methylation profile identifies differentially methylated loci associated with human intervertebral disc degeneration. *PLoS One*.

[114] Monick M. M., Carter A. B., Flaherty D. M., Peterson M. W., Hunninghake G. W. (2000). Protein kinase C zeta plays a central role in activation of the p42/44 mitogen-activated protein kinase by endotoxin in alveolar macrophages. *Journal of Immunology*.

[115] Myouzen K., Kochi Y., Okada Y. (2012). Functional variants in NFKBIE and RTKN2 involved in activation of the NF-*κ*B pathway are associated with rheumatoid arthritis in Japanese. *PLoS Genetics*.

[116] Ni H., Wang X. S., Diener K., Yao Z. (1998). MAPKAPK5, a novel mitogen-activated protein kinase (MAPK)-activated protein kinase, is a substrate of the extracellular-regulated kinase (ERK) and p38 kinase. *Biochemical and Biophysical Research Communications*.

[117] Westhovens R., Keyser F. D., Rekalov D. (2013). Oral administration of GLPG0259, an inhibitor of MAPKAPK5, a new target for the treatment of rheumatoid arthritis: a phase II, randomised, double-blind, placebo-controlled, multicentre trial. *Annals of the Rheumatic Diseases*.

[118] Zotti T., Polvere I., Voccola S., Vito P., Stilo R. (2018). CARD14/CARMA2 signaling and its role in inflammatory skin disorders. *Frontiers in Immunology*.

[119] Kroese L. J., Scheffer P. G. (2014). 8-Hydroxy-2'-deoxyguanosine and cardiovascular disease: a systematic review. *Current Atherosclerosis Reports*.

[120] Wang X. H., Zhu L., Hong X. (2016). Resveratrol attenuated TNF-*α*-induced MMP-3 expression in human nucleus pulposus cells by activating autophagy via AMPK/SIRT1 signaling pathway. *Experimental Biology and Medicine (Maywood, N.J.)*.

[121] Wongpaiboonwattana W., Tosukhowong P., Dissayabutra T., Mutirangura A., Boonla C. (2013). Oxidative stress induces hypomethylation of LINE-1 and hypermethylation of the RUNX3 promoter in a bladder cancer cell line. *Asian Pacific Journal of Cancer Prevention*.

[122] Dowdell J., Erwin M., Choma T., Vaccaro A., Iatridis J., Cho S. K. (2017). Intervertebral disk degeneration and repair. *Neurosurgery*.

[123] Jiang W., Zhang X., Hao J. (2014). SIRT1 protects against apoptosis by promoting autophagy in degenerative human disc nucleus pulposus cells. *Scientific Reports*.

[124] Wang Y., Wu W., Yao C. (2016). Elevated tissue Cr levels, increased plasma oxidative markers, and global hypomethylation of blood DNA in male Sprague-Dawley rats exposed to potassium dichromate in drinking water. *Environmental Toxicology*.

[125] Li X., Phillips F. M., An H. S. (2008). The action of resveratrol, a phytoestrogen found in grapes, on the intervertebral disc. *Spine (Phila Pa 1976)*.

[126] Wuertz K., Quero L., Sekiguchi M. (2011). The red wine polyphenol resveratrol shows promising potential for the treatment of nucleus pulposus-mediated pain in vitro and in vivo. *Spine (Phila Pa 1976)*.

[127] Xia X., Guo J., Lu F., Jiang J. (2015). SIRT1 plays a protective role in intervertebral disc degeneration in a puncture-induced rodent model. *Spine (Phila Pa 1976)*.

[128] Yang S. D., Ma L., Yang D. L., Ding W. Y. (2016). Combined effect of 17*β*-estradiol and resveratrol against apoptosis induced by interleukin-1*β* in rat nucleus pulposus cells via PI3K/Akt/caspase-3 pathway. *PeerJ*.

[129] Krupkova O., Sekiguchi M., Klasen J. (2014). Epigallocatechin 3-gallate suppresses interleukin-1*β*-induced inflammatory responses in intervertebral disc cells in vitro and reduces radiculopathic pain in rats. *European Cells & Materials*.

[130] Cheng Y. H., Yang S. H., Lin F. H. (2011). Thermosensitive chitosan-gelatin-glycerol phosphate hydrogel as a controlled release system of ferulic acid for nucleus pulposus regeneration. *Biomaterials*.

[131] Cheng Y. H., Yang S. H., Liu C. C., Gefen A., Lin F. H. (2013). Thermosensitive hydrogel made of ferulic acid-gelatin and chitosan glycerophosphate. *Carbohydrate Polymers*.

[132] Jeong J. W., Jin C. Y., Kim G. Y. (2010). Anti-inflammatory effects of cordycepin via suppression of inflammatory mediators in BV2 microglial cells. *International Immunopharmacology*.

[133] Nakamura K., Shinozuka K., Yoshikawa N. (2015). Anticancer and antimetastatic effects of cordycepin, an active component of Cordyceps sinensis. *Journal of Pharmacological Sciences*.

[134] Li Y., Li K., Mao L. (2016). Cordycepin inhibits LPS-induced inflammatory and matrix degradation in the intervertebral disc. *PeerJ*.

[135] Xiong X. H., Zhao Y., Ge X. (2011). Production and radioprotective effects of pyrroloquinoline quinone. *International Journal of Molecular Sciences*.

[136] Zhang Q., Ding M., Cao Z., Zhang J., Ding F., Ke K. (2013). Pyrroloquinoline quinine protects rat brain cortex against acute glutamate-induced neurotoxicity. *Neurochemical Research*.

[137] Yang L., Rong Z., Zeng M. (2015). Pyrroloquinoline quinone protects nucleus pulposus cells from hydrogen peroxide-induced apoptosis by inhibiting the mitochondria-mediated pathway. *European Spine Journal*.

[138] Yang S. D., Yang D. L., Sun Y. P. (2015). 17*β*-estradiol protects against apoptosis induced by interleukin-1*β* in rat nucleus pulposus cells by down-regulating MMP-3 and MMP-13. *Apoptosis*.

[139] Anilkumar P., Lu F., Cao L. (2011). Fullerenes for applications in biology and medicine. *Current Medicinal Chemistry*.

[140] Vapa I., Torres V. M., Djordjevic A. (2012). Effect of fullerenol C (60)(OH) (24) on lipid peroxidation of kidneys, testes and lungs in rats treated with doxorubicine. *European Journal of Drug Metabolism and Pharmacokinetics*.

[141] Yang D., Wang D., Shimer A., Shen F. H., Li X., Yang X. (2014). Glutathione protects human nucleus pulposus cells from cell apoptosis and inhibition of matrix synthesis. *Connective Tissue Research*.

[142] Yang X., Jin L., Yao L., Shen F. H., Shimer A. L., Li X. (2014). Antioxidative nanofullerol prevents intervertebral disk degeneration. *International Journal of Nanomedicine*.

[143] Forman H. J., Zhang H., Rinna A. (2009). Glutathione: overview of its protective roles, measurement, and biosynthesis. *Molecular Aspects of Medicine*.

[144] Catenaccio E., Mu W., Lipton M. L. (2016). Estrogen- and progesterone-mediated structural neuroplasticity in women: evidence from neuroimaging. *Brain Structure & Function*.

[145] Cauley J. A. (2015). Estrogen and bone health in men and women. *Steroids*.

[146] Nilsson S., Gustafsson J. Å. (2011). Estrogen receptors: therapies targeted to receptor subtypes. *Clinical Pharmacology and Therapeutics*.

[147] Pöllänen E., Sipilä S., Alen M. (2011). Differential influence of peripheral and systemic sex steroids on skeletal muscle quality in pre- and postmenopausal women. *Aging Cell*.

[148] Liu Q., Wang X., Hua Y. (2019). Estrogen deficiency exacerbates intervertebral disc degeneration induced by spinal instability in rats. *Spine (Phila Pa 1976)*.

[149] Yang S., Zhang F., Ma J., Ding W. (2020). Intervertebral disc ageing and degeneration: the antiapoptotic effect of oestrogen. *Ageing Research Reviews*.

[150] Baret P., Septembre-Malaterre A., Rigoulet M. (2013). Dietary polyphenols preconditioning protects 3T3-L1 preadipocytes from mitochondrial alterations induced by oxidative stress. *The International Journal of Biochemistry & Cell Biology*.

[151] Lei M., Wang J. G., Xiao D. M. (2012). Resveratrol inhibits interleukin 1*β*-mediated inducible nitric oxide synthase expression in articular chondrocytes by activating SIRT1 and thereby suppressing nuclear factor-*κ*B activity. *European Journal of Pharmacology*.

[152] Bastin J., Lopes-Costa A., Djouadi F. (2011). Exposure to resveratrol triggers pharmacological correction of fatty acid utilization in human fatty acid oxidation-deficient fibroblasts. *Human Molecular Genetics*.

[153] Kitada M., Kume S., Imaizumi N., Koya D. (2011). Resveratrol improves oxidative stress and protects against diabetic nephropathy through normalization of Mn-SOD dysfunction in AMPK/SIRT1-independent pathway. *Diabetes*.

[154] Zhou X., Chen M., Zeng X. (2014). Resveratrol regulates mitochondrial reactive oxygen species homeostasis through Sirt3 signaling pathway in human vascular endothelial cells. *Cell Death & Disease*.

[155] Chen M. P., Yang S. H., Chou C. H. (2010). The chondroprotective effects of ferulic acid on hydrogen peroxide-stimulated chondrocytes: inhibition of hydrogen peroxide-induced pro-inflammatory cytokines and metalloproteinase gene expression at the mRNA level. *Inflammation Research*.

[156] Srinivasan M., Sudheer A. R., Menon V. P. (2007). Ferulic acid: therapeutic potential through its antioxidant property. *Journal of Clinical Biochemistry and Nutrition*.

[157] Akagawa M., Nakano M., Ikemoto K. (2016). Recent progress in studies on the health benefits of pyrroloquinoline quinone. *Bioscience, Biotechnology, and Biochemistry*.

[158] Rettberg J. R., Yao J., Brinton R. D. (2014). Estrogen: a master regulator of bioenergetic systems in the brain and body. *Frontiers in Neuroendocrinology*.

[159] Jin L. Y., Lv Z. D., Wang K. (2018). Estradiol alleviates intervertebral disc degeneration through modulating the antioxidant enzymes and inhibiting autophagy in the model of menopause rats. *Oxidative Medicine and Cellular Longevity*.

[160] de Quadros V. P., Tobar N., Viana L. R., Dos Santos R. W., Kiyataka P. H. M., Gomes-Marcondes M. C. C. (2019). The 17*β*-oestradiol treatment minimizes the adverse effects of protein restriction on bone parameters in ovariectomized Wistar rats: relevance to osteoporosis and the menopause. *Bone & Joint Research*.

[161] Andaloussi S. E. L., Mäger I., Breakefield X. O., Wood M. J. (2013). Extracellular vesicles: biology and emerging therapeutic opportunities. *Nature Reviews. Drug Discovery*.

[162] Théry C., Zitvogel L., Amigorena S. (2002). Exosomes: composition, biogenesis and function. *Nature Reviews. Immunology*.

[163] Basit F., van Oppen L. M., Schöckel L. (2017). Mitochondrial complex I inhibition triggers a mitophagy-dependent ROS increase leading to necroptosis and ferroptosis in melanoma cells. *Cell Death & Disease*.

[164] Kim S. U., Park Y. H., Min J. S. (2013). Peroxiredoxin I is a ROS/p38 MAPK-dependent inducible antioxidant that regulates NF-*κ*B-mediated iNOS induction and microglial activation. *Journal of Neuroimmunology*.

[165] Xia C., Zeng Z., Fang B. (2019). Mesenchymal stem cell-derived exosomes ameliorate intervertebral disc degeneration via anti-oxidant and anti-inflammatory effects. *Free Radical Biology & Medicine*.

[166] Zhang J., Zhang J., Zhang Y. (2020). Mesenchymal stem cells-derived exosomes ameliorate intervertebral disc degeneration through inhibiting pyroptosis. *Journal of Cellular and Molecular Medicine*.

[167] Masuda K., An H. S. (2004). Growth factors and the intervertebral disc. *The Spine Journal*.

[168] Gruber H. E., Hoelscher G. L., Ingram J. A., Bethea S., Hanley E. N. (2008). IGF-1 rescues human intervertebral annulus cells from in vitro stress-induced premature senescence. *Growth Factors*.

[169] Wei A., Brisby H., Chung S. A., Diwan A. D. (2008). Bone morphogenetic protein-7 protects human intervertebral disc cells in vitro from apoptosis. *The Spine Journal*.

[170] Ishibashi H., Tonomura H., Ikeda T. (2016). Hepatocyte growth factor/c-met promotes proliferation, suppresses apoptosis, and improves matrix metabolism in rabbit nucleus pulposus cells in vitro. *Journal of Orthopaedic Research*.

